# Longitudinal omics modeling and integration in clinical metabonomics research: challenges in childhood metabolic health research

**DOI:** 10.3389/fmolb.2015.00044

**Published:** 2015-08-05

**Authors:** Peter Sperisen, Ornella Cominetti, François-Pierre J. Martin

**Affiliations:** ^1^GI Health and Microbiome Department, Nestle Institute of Health SciencesLausanne, Switzerland; ^2^Molecular Biomarkers Department, Nestle Institute of Health SciencesLausanne, Switzerland

**Keywords:** metabonomics, empirical modeling, metabolic modeling, longitudinal high dimensional data, clinical phenotype

## Abstract

Systems biology is an important approach for deciphering the complex processes in health maintenance and the etiology of metabolic diseases. Such integrative methodologies will help better understand the molecular mechanisms involved in growth and development throughout childhood, and consequently will result in new insights about metabolic and nutritional requirements of infants, children and adults. To achieve this, a better understanding of the physiological processes at anthropometric, cellular and molecular level for any given individual is needed. In this respect, novel omics technologies in combination with sophisticated data modeling techniques are key. Due to the highly complex network of influential factors determining individual trajectories, it becomes imperative to develop proper tools and solutions that will comprehensively model biological information related to growth and maturation of our body functions. The aim of this review and perspective is to evaluate, succinctly, promising data analysis approaches to enable data integration for clinical research, with an emphasis on the longitudinal component. Approaches based on empirical and mechanistic modeling of omics data are essential to leverage findings from high dimensional omics datasets and enable biological interpretation and clinical translation. On the one hand, empirical methods, which provide quantitative descriptions of patterns in the data, are mostly used for exploring and mining datasets. On the other hand, mechanistic models are based on an understanding of the behavior of a system's components and condense information about the known functions, allowing robust and reliable analyses to be performed by bioinformatics pipelines and similar tools. Herein, we will illustrate current examples, challenges and perspectives in the applications of empirical and mechanistic modeling in the context of childhood metabolic health research.

## Introduction

The rise in chronic and progressive diseases worldwide leads to new challenges in the field of health economics (Nicholson, 2006). The biological complexity of multifactorial disorders such as diabetes, food intolerances, inflammatory diseases, obesity, amongst others, highlights the need to model the web of interactions between genetics, metabolism, environmental factors, lifestyle and nutrition (Nicholson et al., 2011). Furthermore, advances in clinical research pinpoint the critical importance for early diagnosis and treatment of disease progression to minimize their consequences, especially in the case of progressive diseases such as inflammatory bowel diseases or rheumatoid arthritis. Life-long health promotion and disease prevention by nutrition and lifestyle can prevent or delay the onset of chronic diseases (Martin et al., 2012). Identification of personal risk factors for chronic disorders together with a better understanding of individual lifestyle requirements may thus provide a roadmap for a healthier metabolic and clinical status. In such a context, there is a clear need to develop new approaches for enabling personalized therapeutical and nutraceutical management and monitoring solutions (Rezzi et al., 2013; Martin et al., 2013b).

### Systems biology research in health and disease across lifetime

Metabolic syndrome encompasses multifactorial metabolic abnormalities including visceral obesity, glucose intolerance, hypertension, hyperuricaemia, dyslipidemia, and non-alcoholic fatty liver disease, all of which are associated with cardiovascular complications (Mottillo et al., 2010; Scherer et al., 2015). Although insulin resistance (IR) remains a key mechanism underlying the pathophysiology of metabolic syndrome, many studies further investigate the more complex etiology that seems to also depend on genetics, body composition, nutrition, and lifestyle. In particular, adiposity is subject to extensive research, since its quantitative and qualitative (e.g., subcutaneous, visceral) distribution in the body associates with different cardiometabolic, obesogenic, and diabetogenic risks (Wildman et al., 2008). More specifically, epicardial adipose tissue may play an important role for predicting metabolic health in overweight and obese children (Schusterova et al., 2013). Recent multivariate data analyses have associated specific metabolite and lipid profiles to body fat distribution (Wahl et al., 2012; Yamakado et al., 2012; Martin et al., 2013b; Scherer et al., 2015). More specifically, these studies described the close relationships between region-specific fat distribution and the levels of amino acids, sphingomyelin, diacylglycerols, triacylglycerols, and phospholipid species in the blood. Such metabolic insights generate new mechanistic knowledge of complex underlying physiological processes. For instance, the inability of adipose tissue to expand or to store fat, results in lipid overflow to other organs under conditions of excess caloric intake combined with a lack of physical activity (Scherer et al., 2015).

In parallel, new evidence has pointed toward the critical and long-term importance of early nutrition and lifestyle on later health and disease risk predisposition (Koletzko et al., 1998). The rising prevalence of type 2 diabetes and obesity in children is a growing and alarming problem, associated with several short-term and long-term metabolic and cardiovascular complications (Rosenbloom et al., 1999; Marcovecchio and Chiarelli, 2013; Cominetti et al., 2014). Consequently, early identification of people with high risk of becoming diabetic is important because the development of diabetes can be delayed or prevented by lifestyle or medical intervention (Hosking et al., 2014). However, evidence-based dietary guidelines and a more comprehensive characterization of the influence of environmental factors at the onset and during the evolution of type 2 diabetes and obesity are needed (Martin et al., 2013a). As a pre-requisite, reference information on how dietary and lifestyle habits influence metabolic functions must be further expanded. This will enable us to comprehensively document the biological processes associated with individual health at the different stages of the life cycle, including the critical pubertal physiological window, which may appear as a susceptibility period for several metabolic deregulations (Mantovani and Fucic, 2014).

Growth during childhood and adolescence occurs at different rates and is influenced by the interaction amongst genetic, nutritional, and environmental factors, which can lead to different susceptibility to childhood disease and disease risks later in life. This introduces a temporal dimension in the study designs and poses additional analytical challenges. Although little is known about the underlying genetics, growth variability during puberty correlates with a complex genetic architecture linking pubertal height growth, the timing of puberty and childhood obesity and provides new information about processes linking these traits (Cousminer et al., 2013). In the context of metabolic health, childhood and adolescence, obesity introduces a significant disturbance into normal growth and pubertal patterns (Sandhu et al., 2006; Marcovecchio and Chiarelli, 2013). There is evidence in both adults and children that glucose levels that are close to the upper limit of the normal range are indicative of future diabetes. One third of children showing transient hyperglycaemia in the absence of serious illness can be expected to develop diabetes within 1 year (Herskowitz-Dumont et al., 1993; Hosking et al., 2014). IR is associated with diabetes and is modulated by complex patterns of external factors throughout childhood that remains poorly understood. IR is higher during puberty in both males and females, with some studies showing the increase to be independent of changes in adiposity (Jeffery et al., 2012). Modeling of longitudinal data on IR, its relationship to pubertal onset, and interactions with age, sex, adiposity, and IGF-1 has recently been conducted (Jeffery et al., 2012). The study exemplified how IR starts to rise in mid-childhood, some years before puberty, with more than 60% of the variation in IR prior to puberty remaining unexplained. In addition, conventional markers, such as HbA1c, that are used to detect diabetes, or to identify adult individuals at risk of developing diabetes, and for adult metabolic disease risk, are not sensitive and specific enough for pediatric applications, suggesting that other factors influence the variance of these markers in youth (Hosking et al., 2014). One key factor currently being studied is the excess of body weight during childhood which can also influence pubertal development, through an effect on timing of pubertal onset and pubertal hormonal levels (Marcovecchio and Chiarelli, 2013). Additionally, skeletal growth and changes in body composition during growth show important variability in both genders (Ballabriga, 2000). The link between fat and puberty is complex and gender-specific. Body fat of contemporary UK children, for example, does not appear to be deleterious to bone quality (Streeter et al., 2013). Moreover, in girls, higher IR limits further gain in body fat in the long term, an observation consistent with insulin desensitization as an adaptive response to weight gain (Hosking et al., 2011). The complex dynamics of growth and development also involve changes in biological processes that influence basal metabolic function (for instance, resting energy expenditure) and physical activity. The role of resting energy expenditure and weight gain in children is subject to controversy, with particular interest in studying whether low energy expenditure may be a predisposing factor for childhood obesity (Griffiths et al., 1990), and in better understanding of energy requirements prior to and during puberty (Hosking et al., 2010). In recent years advances in microbiota research has provided compelling evidence that the intestinal microbiota contributes to the overall health status of the host and therefore plays an important role in modulating the effect of nutrition on health and disease (Nicholson et al., 2012). In particular, there is increasing evidence for the role that the gut microbiota plays in regulating fat storage and energy homeostasis in the host, hence acting as an important environmental factor for diabetes and obesity (Musso et al., 2010). We and others (Wikoff et al., 2009; Moco et al., 2012, 2014; Tremaroli and Bäckhed, 2012; Sommer and Bäckhed, 2013) have also demonstrated how specific metabolic activities of gut bacterial species can provide the host with new biochemical compounds in sufficient amounts to be detected in the systemic blood stream. These host-gut bacterial co-metabolites may subsequently impact human host metabolism, for instance through modulating quantitatively and qualitatively the nutrient and calories made available to the host throughout digestion (Jumpertz et al., 2011; Martin et al., 2013a).

### Omics modeling and integration in clinical research

The rising prevalence of multifactorial disorders, the lack of understanding of the molecular processes at play, and the need for disease prediction in asymptomatic conditions are some of the many challenges that systems biology is well-suited to address. With its aim to connect the information flow between the different organizational levels of life such as the genome, epigenome, transcriptome, proteome, and metabolome, systems biology approaches are becoming highly relevant for assessing the connection between human physiology and nutrition (Mantovani and Fucic, 2014; Moco et al., 2014). Systems biology also aims at understanding the global dynamics of biological processes to gain a deep understanding of the system, which adds an additional layer of complexity to existing intra-cohort heterogeneities, inter-laboratory methodology differences and changes in the instrumentation (Moco et al., 2014). Omics technologies are often employed to generate a snapshot of the system being studied, at multiple pathway levels, yet only considering cross-sectional information. Therefore, integrative solutions and resources are becoming nowadays a pre-requisite to clinically leverage the knowledge from large amounts of existing omics data collected from different compartments, and ultimately to provide a unified view and personalized therapeutic approaches to disease (Moco et al., 2014). In the context of childhood metabolic studies, major challenges lie in the high dynamics (e.g., metabolic requirements for growth and development), specificities (e.g., hormonal maturation) and amplitude of changes (e.g., acute growth, major switch in the distribution of body fat and lean mass) that affect the biological, physiological, clinical, and anthropometric parameters. Hence, there is a need to adapt methodologies and design of experiment to explore processes related to growth, development, maturation and pubertal stages over months and years of the childhood spectrum.

The aim of this current review and perspective is to evaluate, summarily, some promising data analysis approaches to enable data integration for clinical research, with an emphasis on the longitudinal component (Table 1). Approaches based on empirical (statistics) and mechanistic modeling of omics data are essential to leverage findings from high dimensional omics datasets and enable biological interpretation and clinical translation. Empirical methods are based on direct observations, measurements, and extensive data records. These methods provide quantitative descriptions of patterns in the data and do not attempt to describe underlying processes or the mechanisms involved. Therefore they are mostly used for exploring and mining datasets. Contrasting with empirical approaches, mechanistic models aim at understanding the behavior of a system's components (Thakur, 1991). Mechanistic models are based on the most comprehensive set of available knowledge of the systems of interest (knowledge base)—more than just the data used to train it. They are rooted in two basic principles, namely (i) every observed phenomenon is based on multiple inter-connected processes; and (ii) when the most significant processes are represented mathematically, the simulated output resembles the actual observations. Mechanistic models may also lead to the discovery of emerging properties. These are properties that arise through interactions among smaller or simpler entities but they cannot be observed within the isolated smaller entities. In biology the most prominent mechanistic models are the genome scale metabolic models. They are built on current knowledge (biochemical, metabolic, transcriptional, translation, and signaling) and condense information about the known functions of protein-encoding genes, how these genes/proteins interact with other bioactive compounds and associated reactions, allowing robust and reliable analyses to be performed by bioinformatics pipelines and similar tools (Shen et al., 2010). They are also the base for multi-scale models. In the following sections, we will illustrate current examples, challenges and perspectives in the applications of empirical and mechanistic modeling in the context of childhood metabolic health research.

**Table 1:** **Overview of methods and relevance for childhood metabolic health research**.

**Field of data analysis**	**Main methods**	**Strengths**	**Weaknesses**	**References**	**Relevance for childhood metabolic health**
High dimensional omics data	^*^ PCA ^*^PLS ^*^SVM ^*^RF	^*^ Low n, high p ^*^Linear and non-linear relationships ^*^Suitable for classification ^*^Avoids multiple testing and takes full variance/covariance information into account	^*^ Unable to capture subjects' trajectories ^*^Problematic if there exists autocorrelation between variables/subjects	Geladi and Kowalski, 1986; Wold et al., 2001; Jolliffe, 2002; Montoliu, 2015	^*^ Stratification of childhood group behavior ^*^Prediction early vs late status
Longitudinal omics data	^*^ Mixed models ^*^Markov models ^*^Bayesian models ^*^Timeseries analysis of Lomb-Scargle [Fourier] transformed omics data	^*^ Model complex curves of longitudinal trajectories	^*^ One dataset at a time ^*^Application dependent of experimental design ^*^Problematic when dealing with missing data or low n	Carin et al., 2012; Chen et al., 2012; Cominetti et al., 2014	^*^ Modeling individual childhood trajectories ^*^Predicting/forecasting progression of metabolic readouts
Combined analysis of multiple omics data	^*^ DISCO-SCA ^*^PARAFAC ^*^MCR-ALS ^*^CCA ^*^Clustering of Lomb-Scargle [Fourier] transformed omics data	^*^ Combined analysis of omics data ^*^Relationships within a single or a few blocks at the same time	^*^ How to weight variables when there is a large difference between dimensions p and q	Hotelling, 1936; Montoliu et al., 2009; Martin et al., 2010; Chen et al., 2012; Schouteden et al., 2013	^*^ Signature common or specific to different age/disease groups
Mechanistic models	^*^ ODEs ^*^GEMs	^*^ Describe underlying processes or the mechanisms involved since based on complete metabolic network ^*^Identification of reactions that are causally related to phenotype ^*^Identification of knowledge gaps	^*^ Time consuming to build ^*^Combination of the 3 levels signaling, gene regulation and metabolism still not completely solved ^*^Addition of kinetic information still in its infancy	Bordbar et al., 2011; Goncalves et al., 2013; Mardinoglu et al., 2013	^*^ Generation of testable hypotheses ^*^Mechanistic interpretation of childhood phenotypes

## Integration of longitudinal omics data: methods and challenges

Unlike for adult and elderly population studies, there is a lack of standards and thresholds used to characterize healthy status during childhood, as well as a lack of comprehensive human trials which could guide its study. Moreover, as previously discussed, the nature of growth and development occurring across childhood is linked with complex patterns of dynamics and amplitudes of changes not observed in adult and elderly. Therefore, there is a need to include a wider number of data types including time resolved data and to have a more exploratory type of approach when analyzing the data.

**Figure 1:**
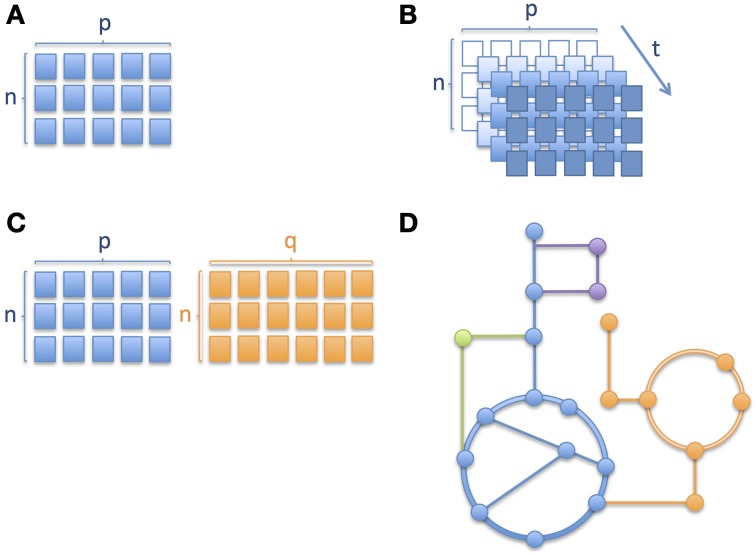
**Different levels of complexity in longitudinal omics data analysis**. Schematic pictures depicting **(A)** a matrix with *n* number of subjects/samples and *p* number of analytes or variables measured, where *n* < *p*, **(B)** several matrices of same variables measured over time, where an increase in color gradient represents a change in time *t;* the variable corresponding to a given time point when the samples were collected or the measurements obtained, **(C)** two matrices of different platforms or variable types (e.g., metabolites and proteins) with different numbers of columns and **(D)** metabolic pathways where nodes correspond to metabolites and edges connecting the nodes correspond to enzymatic reactions. Different colors correspond to different metabolic pathways. In Section Integration of Longitudinal Omics Data: Methods and Challenges we address alternative methods currently used to overcome such complexity.

Similarly to other omics technologies, metabolic profiling (Nicholson et al., 1999; Fiehn, 2002; Smith et al., 2006) based on mass spectrometric (MS) and nuclear magnetic resonance spectroscopy (NMR) produce data, analysis of which brings a number of challenges, with some requiring special attention in clinical omics studies, namely (i) high-dimensional nature of omics data; (ii) longitudinal aspect of multivariate omics data; (iii) multiple omics datasets; and (iv) mechanistic interpretation. The different levels of complexity are depicted through a series of schematic pictures in Figure 1. In the case of childhood metabolic health research these challenges are clearly present and important to address.

### High-dimensional omics data

The high number of variables in omics data involves working with a particular structure between the variables, often related to their analytical or biological relationships, which results in the need for complex frameworks for biomarker discovery (Montoliu, 2015). Furthermore, even if most omics data types are continuous, it is not uncommon to have to deal with discrete variables (clinical or experimental). To address such challenges, multivariate data analyses appears as a more appropriate alternative to the standard approach of univariate analysis plus multiplicity testing correction (Massart et al., 1997; Montoliu, 2015). From the set of techniques driven by a pure chemometric approach, Principal Component Analysis (PCA) (Jolliffe, 2002), Partial Least Squares regression (PLS) (Geladi and Kowalski, 1986; Wold et al., 2001) and their derivates, such as Orthogonal Projection on Latent Structures (OPLS) (Trygg and Wold, 2002, 2003), are amongst the reference methodologies which perform well in low *n* (subjects), high *p* (observations) datasets (where *n* refers to the sample size and *p* to the number of dimensions, as in Figure 1A) through the projection of multivariate data onto a reduced subspace (Richards et al., 2010). PLS methods adapt well to linear and non-linear relationships, but require a validation process to assess whether they apply in a more general way and to minimize overfitting (Westerhuis et al., 2008). Moreover, since PLS assumes a given variable distribution and the linearity of the model, there is an additional need for a careful validation of these features (Montoliu, 2015). Variants of these methodologies are employed when the response variable is categorical (Westerhuis et al., 2010). These approaches are referred to as Linear Discriminant Analysis (LDA), e.g., Partial Least Squares Discriminant Analysis (PLS-DA) (Barker and Rayens, 2003) and Orthogonal Projection on Latent Structures Discriminant Analysis (OPLS-DA) (Bylesjö et al., 2006).

Several other classification algorithms focused on solving low “*n* to *p*” ratio issues (Figure 1A) have been developed, but objective criteria to assess performance and conditions of use remain undefined. It is unlikely that a universal classifier/regressor can satisfy all conditions, and therefore applications of the different methodologies are driven by the research question (Montoliu, 2015). As extensively discussed by Gomez-Cabrero et al., the analysis of large and heterogeneous data sets encourage researchers to develop novel data integration methodologies (Gomez-Cabrero et al., 2014). Amongst these methodologies, machine learning approaches or regularized statistical methods provide a wealth of tools that can learn from and make predictions on data (classification and regression), including Support Vector Machines (SVMs), Random Forests (RFs) and Multilayer perceptrons on the one side; and SPLS, Lasso or Elastic Nets (ENs) on the other side. However, the use of kernels and weight connection layers in Multilayer perceptrons removes any traceability of the role of the individual variables in the model (Montoliu, 2015).

In the context of childhood research, such approaches remain very relevant, allowing to compare groups of subjects, similarly to what is applied when studying obesity, overweight, diabetes, impaired insulin, and glucose control in adults, as exemplified by Wahl et al. (2012). Moreover, methodologies like RFs or PLS, are extremely useful to predict the influence of early metabolic status on later outcomes. However their application in the context of time resolved data remains more challenging as discussed hereafter.

### Longitudinal multivariate omics data

When it comes to longitudinal omics data, i.e., one or more type of omics data measured over time (see also Figure 1B where the same matrix of measurements is repeated at different time points, depicted using an increase in intensity of color), the statistical analysis becomes even more challenging (Dean et al., 2009; Stanberry et al., 2013; Cominetti et al., 2014). However, longitudinal studies are key to understand the global evolution of biological processes. Such studies aim typically at following populations of subjects over time. Resulting time profiles can be clustered to identify subgroups or can be used for monitoring, forecasting and diagnostic purposes (Albert and Schisterman, 2012; Liquet et al., 2012). In addition, the time dimension is important and often specific to the type of data and clinical endpoints in human trials, ranging from minutes and hours, to months and even years. Indeed, the biological processes described by the omics data show specific time-dependent modulation, amplitude of change and regulatory mechanisms; for instance, gene expression and metabolites involved in gluconeogenesis show very different and specific time scale but contribute to the same biochemical processes. Moreover, repeated measurements are often unequally-spaced in time (in our pictorial representation of Figure 1B, this would mean that the colors of the cells of the matrices do not change in a linear manner) and it is important to account for this difference in the model (Albert and Schisterman, 2012) as well as for delays in time-to-event such as disease onset or phenotypic change. Additional challenges when dealing with longitudinal data are auto-correlation of repeated measurements of the same variables, random effects, missing data, and dropouts, which are being discussed hereafter (Dean et al., 2009; Carin et al., 2012). Auto-correlation can be both a limitation and an advantage depending on the type of analysis. For instance, it is a limitation when trying to use certain techniques such as projection-based methods (e.g., PCA, PLS) which are well suited to tackle high-dimensional datasets but that do not take into account subjects' trajectories. With respect to missing data and dropouts it is important to assess if the reason for the data missing is related to the process under observation or not (Albert and Schisterman, 2012). One approach to deal with missing data could be to impute it while avoiding biased results.

Despite all the challenges mentioned above, the analysis of longitudinal omics datasets typically provides major advantages, not only in terms of gain in information, but also through (i) an increase in the statistical power of the studies (Zeger and Liang, 1992), (ii) a decrease in noise (if correlations of repeated measurements and inter individual variability are properly accounted for) (Liquet et al., 2012; Cominetti et al., 2014), as well as (iii) an increase in the robustness to model specification (Zeger and Liang, 1992).

A range of solutions have been proposed, including Generalized Linear Mixed Models (GLMM), Generalized Estimating Equations (GEE), Markov models, non-parametric or semi-parametric models or Bayesian models, factor analysis, dictionary learning, dynamical pathway analysis, latent growth curves, amongst others (Carin et al., 2012; Stanberry et al., 2013; Cominetti et al., 2014). Alternatively to those parametric methods, non-parametric or semi-parametric statistical models remain widely employed, being more flexible than parametric models, to model the complex curves of longitudinal trajectories (Dean et al., 2009), especially when the variations in the omics variables are large or are induced by major biological events (e.g., changes in metabolism and requirements during the growth of the child, puberty and the onset and/or remission of a disease). However, considering the vast array of techniques and their specific advantages and limitations, it will often depend on the overall objective of the study, and constraints imposed by the data, when choosing the best adapted modeling tools. Up to this point we were considering one data set generated over time. This can be further extended to multiple omics datasets, like the ones represented in Figure 1C.

### Combined analysis of multiple multivariate datasets

In addition to modeling temporal omics data, combined analysis of different omics data sets is still in its infancy (Gomez-Cabrero et al., 2014). As clearly presented by Gomez-Cabrero et al., the term of data integration refers to the integrative study of different sources and types of data from a given system (Gomez-Cabrero et al., 2014). In this context, identifying shared or common information among two or more sets of data from a biological process under study can help us to better describe underlying molecular events. However, these large heterogeneous data sets result in some significant challenges (Gomez-Cabrero et al., 2014). First, the fundamental differences in the data types need to be considered, including the difference in their variance-covariance structure, the multi-scale nature of omics data and differences in sizes of omics datasets (see also Figure 1C showing two matrices with sizes *n* by *p* and *n* by *q* respectively, where *p* and *q* are the different number of analytes measured), which brings the issue of having to weight groups of variables differently. Richards et al. have previously summarized key approaches for intra- and inter-omic fusion strategies in a metabonomics-driven context (Richards et al., 2010). Their work highlighted some promising methods for inter-instrument, inter-sample type and inter-omics integration, namely multiblock hierarchical PCA, consensus PCA, Parallel Factor Analysis (PARAFAC), Multivariate Curve Resolution-Alternative Least Squares (MCR-ALS) and O_2_PLS techniques. MCR-ALS and PARAFAC are well adapted to assess functional relationships across matrices and to enable the characterization of compartment-specific metabolic signatures (Montoliu et al., 2009; Martin et al., 2010). Eventually, such approaches are also relevant for stepwise variable and data-block selection for further multivariate and longitudinal analysis. From other related fields, such as ecology and multi-species genomics, a variety of methodologies are being used to enable various data integration strategies, including Generalized Singular Value Decomposition (GSVD), Latent Variable Multivariate Regression (LVMR), Simultaneous Component Analysis (SCA), Canonical Correlation Analysis (CCA) (Hotelling, 1936), Co-Inertia Analysis (COIA), Integrative Bi-Clustering or Multiple Factor Analysis (MFA). These approaches may also offer novel opportunities in the field of clinical metabonomics. Moreover, with the aim of identifying common and data-specific information for a given omics data set, methods based on two-block latent variable regression with an integral OSC filter, such as O2PLS (Trygg and Wold, 2002, 2003) are being used (especially in the field of Metabonomics), but Joint and Individual Variation Explained (JIVE) (Lock et al., 2013) and DIStinct COmmon SCA (DISCO-SCA) (Schouteden et al., 2013) may offer some advantages in terms of analytical strategies. JIVE represents an extension of PCA, it works by decomposing data into three elements, one of which captures the joint structure between data types, another captures structure individual to each data type and a third element which captures the residual noise (Lock et al., 2013). JIVE may offer advantages compared to CCA and PLS approaches and it could offer some promising capabilities for the integrated analysis of omics data (Lock et al., 2013). SCA methods are well adapted to study linked data and model a small number of simultaneous components that maximally account for the variations in the data sets (Schouteden et al., 2013). While SCA reflects a mix of common and distinct information, the DISCO-SCA approach aims at solving this problem in multi-block data analysis, by enabling both the modeling of relationships across all the data types under consideration, but also to explore the relationships within a single or a few selected blocks at the same time (Schouteden et al., 2013). Schouteden et al. presented an example where children from different age groups are given the same personality questionnaire, which results in a set of child-by-item data blocks, with each data block pertaining to a specific age group and with the different data blocks having the questionnaire items in common. DISCO-SCA could enable the analysis of both general personality dimensions and dimensions that are specific for a certain developmental stage. In the context of metabolic phenotype in childhood, such a method could thus allow the study of molecular processes related to growth, and the simultaneous exploration of age-specific phenotype.

The integrative personal omics profile (iPOP) analysis (Chen et al., 2012) tries to go one step further, namely combining multiple time-resolved multivariate datasets, such as genomics, transcriptomics, proteomics, and metabolomics profiles from a single individual measured over a 14-month period. The datasets were first transformed using a Lomb–Scargle (Lomb, 1976; Scargle, 1982, 1989) [Fourier] transformation in order to remove the effect of uneven data sampling in time. Based on this transformed data, the original time-series were reconstructed using an inverse Fourier transform and evenly resampling frequencies/times (for more details see also Chen et al., 2012 and references therein). This allowed the use of standard time-series analysis methods and the clustering of the combined datasets. As a proof-of-concept, the longitudinal iPOP study has shown the potential to interpret healthy and disease status by connecting genomic information with additional dynamic omics activity. These methodologies could offer unprecedented opportunities to further explore the functional relationships between omics biological data and growth and development, and subsequently to allow novel characterizations of factors contributing to a healthy or unhealthy childhood trajectory.

However, these methods provide quantitative descriptions of patterns in the data and do not attempt to describe underlying processes or the mechanisms involved. In this respect mechanistic models are an important addition to empirical data analysis providing a framework to mathematically represent current biological knowledge as well as data interpretation and clinical translation of the multiple cellular processes captured by the omics approaches.

### Mechanistic and biological interpretation of models based on omics data

In the last couple of years mechanistic modeling has become more and more popular as approach to model phenotypes under different conditions and therefore to expand the understanding of complex biological systems. In contrast to the previously discussed methods that try to develop models based on the given data, mechanistic models are knowledge–based models and are therefore independent of the data. At the lower end of the modeling hierarchy, in terms of biological organization, we have the cell. Their phenotype is mainly controlled at the following three levels: (i) metabolism: enzyme-catalyzed chemical transformations taking place in a cell that either consumes metabolites for energy production or generates small molecules that serve as building blocks, (ii) gene regulation: control of increase or decrease of transcripts (mRNA) and their translation into proteins, and (iii) signaling: complex communication system that combines proteins, lipids, and small molecules in various ways allowing cells to sense the environment and respond correctly. These three levels are linked through diverse types of interactions but with respect to modeling they are still mostly treated separately using mathematical formalisms that are specific to the level that is modeled and that reflect the molecules and the processes involved. Combined models are still rare, since there is currently no single modeling formalism that can deal with the different biological aspects.

Ordinary differential equations (ODEs) are frequently used to describe metabolic pathways. However, the challenge with ODEs is that it is often difficult to obtain the parameters required for the model. Consequently, when it comes to genome-scale models and simulations they quickly become unfeasible. Alternatively, for larger networks, genome-scale metabolic models (GEMs) or Boolean networks are widely used (Goncalves et al., 2013). In particular, constraint-based GEMs are well suited to handle the complexity of the cellular metabolism leading to a better understanding of the full cellular metabolism at the systems level (Figure 1D depicts metabolic pathways with the nodes representing metabolites and solid edges representing enzymatic reactions). Therefore GEMs are very useful to study disorders that have a strong metabolic component.

Currently most GEMs are based on steady-state analysis. Only recently different groups have started with the construction of kinetic models (Chakrabarti et al., 2013; Stanford et al., 2013). However, in absence of real data, estimation of the kinetic parameters remains a challenge. Consequently they are not yet used for higher eukaryotes.

The starting point for the generation of GEMs for human cells are essentially two generic literature-based GEMs, Recon 1 (Duarte et al., 2007) and the Edinburgh Human Metabolic Network (EHMN) (Ma et al., 2007), which have been developed by different research groups with the aim to study human metabolism. These generic GEMs were later merged into one database, the Human Metabolic Reaction (HMR) database (Agren et al., 2012), together with reactions related to human metabolism from KEGG (Kanehisa et al., 2010). Recently the HMR database has been updated with data from Reactome (Croft et al., 2011), HepatoNet1 (Gille et al., 2010), Lipidomics Gateway (Harkewicz and Dennis, 2011) and the HumanCyc database (Romero et al., 2005).

Currently, several cell/tissue-specific GEMs for liver (hepatocytes) (Gille et al., 2010; Jerby et al., 2010), kidney (Chang et al., 2010), brain (3 neuron types and astrocytes) (Lewis et al., 2010), alveolar macrophage (Bordbar et al., 2010), cardiomyocyte (Karlstadt et al., 2012), adipocyte (Mardinoglu et al., 2013), multi-tissue models (hepatocytes, myocytes, and adipocytes) (Bordbar et al., 2011) or whole organelles, such as mitochondria (Aimar-Beurton et al., 2002; Thiele et al., 2005) are publicly available.

In the last couple of years the cost of large-scale omics data generation has considerably decreased but analyzing and more specifically the interpretation of such data remains a challenge. This is mainly due to the complexity of the underlying cellular processes which involve the regulation of multiple genes that are not fully understood in terms of function and interactions amongst them (Palsson and Zengler, 2010). In an attempt to overcome these challenges, several groups introduced the use of GEMs to place omics data in the context of the cellular metabolism (Palsson, 2009; Yizhak et al., 2010; Ideker and Krogan, 2012). GEMs can be reconstructed based on high-throughput omics data, but they also serve as a computational framework to analyze and interpret such data as a network where the nodes represent the substrates/products and the edges the reactions, like the schematic representation in Figure 1D. These networks are then transformed into stoichiometric matrices which serve as the base for constraint-based modeling (Famili et al., 2003), into which numerical omics data can be effectively plugged. Moreover, personalized GEMs can be reconstructed in the same way as cell/tissue-specific models are generated. In this case one would start from omics data obtained from a single patient and earlier studies on inborn errors of metabolism (Shlomi et al., 2009) and metastatic breast cancer (Jerby et al., 2012) show that GEMs are of potential use in the discovery of biomarkers.

A multi-cellular/multi-tissue type GEM was elegantly described by Bordbar et al. (2011). They developed a model that connects GEMs that represent human adipocytes, hepatocytes and myocytes and hence allows connecting the metabolic pathways of the three cell types. They used the resulting model to study diabetes and in this respect they simulated the behavior of known cross-cell metabolic cycles. In order to study differences in the metabolic activity between obese and obese type II patients that underwent a gastric bypass surgery high-throughput data was integrated with the multi-cellular/multi-tissue type GEM. This approach allowed the authors to link known physiological changes seen in these patients with a mechanistic understanding. These findings can be described as emergent properties, since they could only be observed using the multi-tissue modeling approach. It would not have been possible to make these observations from transcription data only. This example illustrates how such approaches could be used to obtain a mechanistic understanding of the phenotypic evolution during childhood by linking the phenotype with the underlying metabolism.

The multi-cellular/multi-tissue study described above essentially connects GEMs that represent different cell types. Such multiscale models are ideal to model biological systems, since biological systems are intrinsically complex; composed of multiple functional networks, which operate across different temporal and spatial levels to maintain growth, development, and reproduction. Multiscale models are combinations of continuous and discrete modeling strategies either deterministic or stochastic. Such computational models are uniquely positioned to capture the connectivity between these divergent scales of biological function, as they can bridge the gap in understanding between isolated *in vitro* experiments and whole-organism *in vivo* models. Starting at the cell level, the next step would be to combine GEMs using an agent based modeling approach to represent cell networks and tissues. These tissues would then need to be combined into larger, whole-organ models, typically using finite element approaches (Moreno et al., 2011). However, no single comprehensive gene-to-organism multiscale model has been developed so far but remains subject to intensive research (Walpole et al., 2013).

Successful applications of GEMs may lead to the generation of testable hypotheses with strong mechanistic interpretations and identification of knowledge gaps. Moreover GEMs may lead to the prediction of proteins and/or metabolites that are key in the evolution of a disease and provide a context-dependent framework for the analysis of disease specific omics data. Consequently GEMs can be used to better understand the relationship between genotype and phenotype and generate new biological knowledge (Patil and Nielsen, 2005; Lewis et al., 2012), possibly leading to the discovery of biomarkers; drug targets and new therapeutic agents (Jerby and Ruppin, 2012; Mardinoglu and Nielsen, 2012).

## Conclusion

Systems biology methodologies will help better understanding the molecular mechanisms involved in growth and development through childhood, and consequently will result in new insights about metabolic and nutritional requirements of infants, children and adults. To achieve this, a better deciphering of the physiological processes at an anthropometric, cellular and molecular level for any given individual is needed. In this respect, novel omics technologies in combination with sophisticated data modeling techniques are key, as summarized in Table 1. Amongst the major challenges when integrating longitudinal omics data are the high dimensional nature of the omics data, the longitudinal aspect of multivariate omics data and integrating multiple datasets, as well as the mechanistic interpretation of the omics data. Projection methodologies such as PCA and PLS work well for low n, high p datasets, but not for longitudinal data. Therefore, methodologies able to adapt to the complexity of individual trajectories are needed, such as non-parametric statistical models, GEE, Markov models, Factor analysis and Bayesian models that have appeared as good tools for modeling longitudinal data. Furthermore, the integration of different omics datasets could be achieved via techniques including CCA, COIA, multiple factor analysis and integrative biclustering. Some of these tools utilize a multi-block approach and/or study the covariance between the different matrices. In contrast to these empirical approaches mechanistic modeling has become a key methodology to better understand biological systems. In the last couple of years, these methods have made big progresses and can be used as framework to interpret omics data. Also, such models serve as knowledge bases that combine the current understanding in a mathematical form and allow to make phenotypic predictions under different conditions and to identify gaps. However, due to the high complexity of the network of influential factors determining individual trajectories, the field is still in its infancy and it becomes imperative to develop proper tools and solutions that will comprehensively model biological information related to growth and maturation of our body functions.

## Conflict of interest statement

The authors declare that the research was conducted in the absence of any commercial or financial relationships that could be construed as a potential conflict of interest.

## References

[B1] Agren R. Bordel S. Mardinoglu A. Pornputtapong N. Nookaew I. Nielsen J. (2012). Reconstruction of genome-scale active metabolic networks for 69 human cell types and 16 cancer types using INIT. PLoS Comput. Biol. 8:e1002518. 10.1371/journal.pcbi.1002518 22615553 PMC3355067

[B2] Aimar-Beurton M. Korzeniewski B. Letellier T. Ludinard S. Mazat J. P. Nazaret C. (2002). Virtual mitochondria: metabolic modelling and control. Mol. Biol. Rep. 29, 227–232. 10.1023/A:1020338115406 12241062

[B3] Albert P. S. Schisterman E. F. (2012). Novel statistical methodology for analyzing longitudinal biomarker data. Stat. Med. 31, 2457–2460. 10.1002/sim.5500 22969022 PMC3462351

[B4] Ballabriga A. (2000). Morphological and physiological changes during growth: an update. Eur. J. Clin. Nutr. 54(Suppl. 1), S1–S6. 10.1038/sj.ejcn.1600976 10805030

[B5] Barker M. Rayens W. (2003). Partial least squares for discrimination. J. Chemom. 17, 166–173. 10.1002/cem.785

[B6] Bordbar A. Feist A. M. Usaite-Black R. Woodcock J. Palsson B. O. Famili I. (2011). A multi-tissue type genome-scale metabolic network for analysis of whole-body systems physiology. BMC Syst. Biol. 5:180. 10.1186/1752-0509-5-180 22041191 PMC3219569

[B7] Bordbar A. Lewis N. E. Schellenberger J. Palsson B. O. Jamshidi N. (2010). Insight into human alveolar macrophage and M. tuberculosis interactions via metabolic reconstructions. Mol. Syst. Biol. 6, 422. 10.1038/msb.2010.68 20959820 PMC2990636

[B8] Bylesjö M. Rantalainen M. Cloarec O. Nicholson J. K. Holmes E. Trygg J. (2006). OPLS discriminant analysis: combining the strengths of PLS-DA and SIMCA classification. J. Chemom. 20, 341–351. 10.1002/cem.1006

[B9] Carin L. Hero A. III. Lucas J. Dunson D. Chen M. Heñao R.. (2012). High-dimensional longitudinal genomic data: an analysis used for monitoring viral infections. IEEE Signal Process. Mag. 29, 108–123. 10.1109/MSP.2011.943009 24678238 PMC3964679

[B10] Chakrabarti A. Miskovic L. Soh K. C. Hatzimanikatis V. (2013). Towards kinetic modeling of genome-scale metabolic networks without sacrificing stoichiometric, thermodynamic and physiological constraints. Biotechnol. J. 8, 1043–1057. 10.1002/biot.201300091 23868566

[B11] Chang R. L. Xie L. Xie L. Bourne P. E. Palsson B. Ø. (2010). Drug off-target effects predicted using structural analysis in the context of a metabolic network model. PLoS Comput. Biol. 6:e1000938. 10.1371/journal.pcbi.1000938 20957118 PMC2950675

[B12] Chen R. Mias G. I. Li-Pook-Than J. Jiang L. Lam H. Y. Chen R.. (2012). Personal omics profiling reveals dynamic molecular and medical phenotypes. Cell 148, 1293–1307. 10.1016/j.cell.2012.02.009 22424236 PMC3341616

[B13] Cominetti O. Collino S. Martin F. P. (2014). Monitoring metabolism across childhood: biomarkers for nutritional health and disease risk management. Agro. Food Ind. Hi Tech. 25, 14–18.

[B14] Cousminer D. L. Berry D. J. Timpson N. J. Ang W. Thiering E. Byrne E. M.. (2013). Genome-wide association and longitudinal analyses reveal genetic loci linking pubertal height growth, pubertal timing and childhood adiposity. Hum. Mol. Genet. 22, 2735–2747. 10.1093/hmg/ddt104 23449627 PMC3674797

[B15] Croft D. O'kelly G. Wu G. Haw R. Gillespie M. Matthews L.. (2011). Reactome: a database of reactions, pathways and biological processes. Nucleic Acids Res. 39, D691–D697. 10.1093/nar/gkq1018 21067998 PMC3013646

[B16] Dean C. L. X. Neuhaus J. Wang L. Wu L. Yi G. (2009). Workshop on Emerging Issues in the Analysis of Longitudinal Data. Banff, AB: Banff International Research Station (BIRS).

[B17] Duarte N. C. Becker S. A. Jamshidi N. Thiele I. Mo M. L. Vo T. D.. (2007). Global reconstruction of the human metabolic network based on genomic and bibliomic data. Proc. Natl. Acad. Sci. U.S.A. 104, 1777–1782. 10.1073/pnas.0610772104 17267599 PMC1794290

[B18] Famili I. Forster J. Nielsen J. Palsson B. O. (2003). Saccharomyces cerevisiae phenotypes can be predicted by using constraint-based analysis of a genome-scale reconstructed metabolic network. Proc. Natl. Acad. Sci. U.S.A. 100, 13134–13139. 10.1073/pnas.2235812100 14578455 PMC263729

[B19] Fiehn O. (2002). Metabolomics - The link between genotypes and phenotypes. Plant Mol. Biol. 48, 155–171. 10.1023/A:1013713905833 11860207

[B20] Geladi P. Kowalski B. R. (1986). Partial least-squares regression: a tutorial. Anal. Chim. Acta 185, 1–17. 10.1016/0003-2670(86)80028-9

[B21] Gille C. Bölling C. Hoppe A. Bulik S. Hoffmann S. Hubner K.. (2010). HepatoNet1: a comprehensive metabolic reconstruction of the human hepatocyte for the analysis of liver physiology. Mol. Syst. Biol. 6, 411. 10.1038/msb.2010.62 20823849 PMC2964118

[B22] Gomez-Cabrero D. Abugessaisa I. Maier D. Teschendorff A. Merkenschlager M. Gisel A.. (2014). Data integration in the era of omics: current and future challenges. BMC Syst. Biol. 8(Suppl. 2), I1. 10.1186/1752-0509-8-S2-I1 25032990 PMC4101704

[B23] Gonçalves E. Bucher J. Ryll A. Niklas J. Mauch K. Klamt S.. (2013). Bridging the layers: towards integration of signal transduction, regulation and metabolism into mathematical models. Mol. Biosyst. 9, 1576–1583. 10.1039/c3mb25489e 23525368

[B24] Griffiths M. Payne P. R. Stunkard A. J. Rivers J. P. Cox M. (1990). Metabolic rate and physical development in children at risk of obesity. Lancet 336, 76–78. 10.1016/0140-6736(90)91592-X 1975323

[B25] Harkewicz R. Dennis E. A. (2011). Applications of mass spectrometry to lipids and membranes. Annu. Rev. Biochem. 80, 301–325. 10.1146/annurev-biochem-060409-092612 21469951 PMC3410560

[B26] Herskowitz-Dumont R. Wolfsdorf J. I. Jackson R. A. Eisenbarth G. S. (1993). Distinction between transient hyperglycemia and early insulin-dependent diabetes mellitus in childhood: a prospective study of incidence and prognostic factors. J. Pediatr. 123, 347–354. 10.1016/S0022-3476(05)81731-7 8355109

[B27] Hosking J. Henley W. Metcalf B. S. Jeffery A. N. Voss L. D. Wilkin T. J. (2010). Changes in resting energy expenditure and their relationship to insulin resistance and weight gain: a longitudinal study in pre-pubertal children (EarlyBird 17). Clin. Nutr. 29, 448–452. 10.1016/j.clnu.2010.01.002 20138693

[B28] Hosking J. Metcalf B. S. Jeffery A. N. Streeter A. J. Voss L. D. Wilkin T. J. (2014). Divergence between HbA1c and fasting glucose through childhood: implications for diagnosis of impaired fasting glucose (Early Bird 52). Pediatr. Diabetes 15, 214–219. 10.1111/pedi.12082 25705748

[B29] Hosking J. Metcalf B. S. Jeffery A. N. Voss L. D. Wilkin T. J. (2011). Direction of causality between body fat and insulin resistance in children–a longitudinal study (EarlyBird 51). Int. J. Pediatr. Obes. 6, 428–433. 10.3109/17477166.2011.608800 21867370

[B30] Hotelling H. (1936). Relations between two sets of variates. Biometrika 28, 321–377. 10.1093/biomet/28.3-4.321

[B31] Ideker T. Krogan N. J. (2012). Differential network biology. Mol. Syst. Biol. 8, 565. 10.1038/msb.2011.99 22252388 PMC3296360

[B32] Jeffery A. N. Metcalf B. S. Hosking J. Streeter A. J. Voss L. D. Wilkin T. J. (2012). Age before stage: insulin resistance rises before the onset of puberty: a 9-year longitudinal study (EarlyBird 26). Diabetes Care 35, 536–541. 10.2337/dc11-1281 22279034 PMC3322712

[B33] Jerby L. Ruppin E. (2012). Predicting drug targets and biomarkers of cancer via genome-scale metabolic modeling. Clin. Cancer Res. 18, 5572–5584. 10.1158/1078-0432.CCR-12-1856 23071359

[B34] Jerby L. Shlomi T. Ruppin E. (2010). Computational reconstruction of tissue-specific metabolic models: application to human liver metabolism. Mol. Syst. Biol. 6, 401. 10.1038/msb.2010.56 20823844 PMC2964116

[B35] Jerby L. Wolf L. Denkert C. Stein G. Y. Hilvo M. Oresic M.. (2012). Metabolic associations of reduced proliferation and oxidative stress in advanced breast cancer. Cancer Res. 72, 5712–5720. 10.1158/0008-5472.CAN-12-2215 22986741

[B36] Jolliffe I. T. (2002). Principal Component Analysis. New York, NY: Springer-Verlag.

[B37] Jumpertz R. Le D. S. Turnbaugh P. J. Trinidad C. Bogardus C. Gordon J. I.. (2011). Energy-balance studies reveal associations between gut microbes, caloric load, and nutrient absorption in humans. Am. J. Clin. Nutr. 94, 58–65. 10.3945/ajcn.110.010132 21543530 PMC3127503

[B38] Kanehisa M. Goto S. Furumichi M. Tanabe M. Hirakawa M. (2010). KEGG for representation and analysis of molecular networks involving diseases and drugs. Nucleic Acids Res. 38, D355–D360. 10.1093/nar/gkp896 19880382 PMC2808910

[B39] Karlstädt A. Fliegner D. Kararigas G. Ruderisch H. S. Regitz-Zagrosek V. Holzhütter H. G. (2012). CardioNet: a human metabolic network suited for the study of cardiomyocyte metabolism. BMC Syst. Biol. 6:114. 10.1186/1752-0509-6-114 22929619 PMC3568067

[B40] Koletzko B. Aggett P. J. Bindels J. G. Bung P. Ferré P. Gil A.. (1998). Growth, development and differentiation: a functional food science approach. Br. J. Nutr. 80(Suppl. 1), S5–S45. 10.1079/bjn19980104 9849353

[B41] Lewis N. E. Nagarajan H. Palsson B. O. (2012). Constraining the metabolic genotype-phenotype relationship using a phylogeny of in silico methods. Nat. Rev. Microbiol. 10, 291–305. 10.1038/nrmicro2737 22367118 PMC3536058

[B42] Lewis N. E. Schramm G. Bordbar A. Schellenberger J. Andersen M. P. Cheng J. K.. (2010). Large-scale in silico modeling of metabolic interactions between cell types in the human brain. Nat. Biotechnol. 28, 1279–1285. 10.1038/nbt.1711 21102456 PMC3140076

[B43] Liquet B. Le Cao K. A. Hocini H. Thiébaut R. (2012). A novel approach for biomarker selection and the integration of repeated measures experiments from two assays. BMC Bioinformatics 13:325. 10.1186/1471-2105-13-325 23216942 PMC3627901

[B44] Lock E. F. Hoadley K. A. Marron J. S. Nobel A. B. (2013). Joint and Individual Variation Explained (Jive) for integrated analysis of multiple data types. Ann. Appl. Stat. 7, 523–542. 10.1214/12-AOAS597 23745156 PMC3671601

[B45] Lomb N. R. (1976). Least-squares frequency analysis of unequally spaced data. Astrophys. Space Sci. 39, 447–462. 10.1007/BF00648343

[B46] Ma H. Sorokin A. Mazein A. Selkov A. Selkov E. Demin O.. (2007). The Edinburgh human metabolic network reconstruction and its functional analysis. Mol. Syst. Biol. 3, 135. 10.1038/msb4100177 17882155 PMC2013923

[B47] Mantovani A. Fucic A. (2014). Puberty dysregulation and increased risk of disease in adult life: possible modes of action. Reprod. Toxicol. 44, 15–22. 10.1016/j.reprotox.2013.06.002 23791931

[B48] Marcovecchio M. L. Chiarelli F. (2013). Obesity and growth during childhood and puberty. World Rev. Nutr. Diet. 106, 135–141. 10.1159/000342545 23428692

[B49] Mardinoglu A. Agren R. Kampf C. Asplund A. Nookaew I. Jacobson P.. (2013). Integration of clinical data with a genome-scale metabolic model of the human adipocyte. Mol. Syst. Biol. 9, 649. 10.1038/msb.2013.5 23511207 PMC3619940

[B50] Mardinoglu A. Nielsen J. (2012). Systems medicine and metabolic modelling. J. Intern. Med. 271, 142–154. 10.1111/j.1365-2796.2011.02493.x 22142312

[B51] Martin F. P. Collino S. Rezzi S. Kochhar S. (2012). Metabolomic applications to decipher gut microbial metabolic influence in health and disease. Front. Physiol. 3:113. 10.3389/fphys.2012.00113 22557976 PMC3337463

[B52] Martin F. P. Moco S. Montoliu I. Collino S. Da S. L. Rezzi S.. (2013a). *Impact of* breast-feeding, high- and low-protein formula on the metabolism and growth of infants from overweight and obese mothers. Pediatr. Res. 75, 535–543. 10.1038/pr.2013.250 24375085

[B53] Martin F. P. Montoliu I. Collino S. Scherer M. Guy P. Tavazzi I.. (2013b). Topographical body fat distribution links to amino acid and lipid metabolism in healthy non-obese women. PLoS ONE 8:e73445. 10.1371/journal.pone.0073445 24039943 PMC3770640

[B54] Martin F. P. Montoliu I. Kochhar S. Rezzi S. (2010). Chemometric strategy for modeling metabolic biological space along the gastrointestinal tract and assessing microbial influences. Anal. Chem. 82, 9803–9811. 10.1021/ac102015n 21033673

[B55] Massart D. L. Vandeginste B. G. M. Buydens L. M. C. De Jong S. Lewi P. J. Smeyers-Verbeke J. (1997). Handbook of Chemometrics and Qualimetrics. Amsterdam: Elsevier Science B.V.

[B56] Moco S. Candela M. Chuang E. Draper C. Cominetti O. Montoliu I.. (2014). Systems biology approaches for inflammatory bowel disease: emphasis on gut microbial metabolism. Inflamm. Bowel Dis. 20, 2104–2114. 10.1097/MIB.0000000000000116 25029616

[B57] Moco S. Martin F. P. Rezzi S. (2012). A metabolomics view on gut microbiome modulation by polyphenol-rich foods. J. Proteome Res. 11, 4781–4790. 10.1021/pr300581s 22905879

[B58] Montoliu I. (2015). Adopting multivariate non-parametric tools to determine genotype-phenotype interactions in health and disease, in Metabonomics and Gut Microbiota in Nutrition and Disease, eds Kochhar S. Martin F.-P. (London: Springer-Verlag), 45–62.

[B59] Montoliu I. Martin F. P. Collino S. Rezzi S. Kochhar S. (2009). Multivariate modeling strategy for intercompartmental analysis of tissue and plasma (1)H NMR Spectrotypes. J. Proteome Res. 8, 2397–2406. 10.1021/pr8010205 19317465

[B60] Moreno J. D. Zhu Z. I. Yang P. C. Bankston J. R. Jeng M. T. Kang C. (2011). A computational model to predict the effects of class I anti-arrhythmic drugs on ventricular rhythms. Sci. Transl. Med. 3, 98ra83. 10.1126/scitranslmed.3002588 PMC3328405 21885405

[B61] Mottillo S. Filion K. B. Genest J. Joseph L. Pilote L. Poirier P.. (2010). The metabolic syndrome and cardiovascular risk a systematic review and meta-analysis. J. Am. Coll. Cardiol. 56, 1113–1132. 10.1016/j.jacc.2010.05.034 20863953

[B62] Musso G. Gambino R. Cassader M. (2010). Obesity, diabetes, and gut microbiota: the hygiene hypothesis expanded? Diabetes Care 33, 2277–2284. 10.2337/dc10-0556 20876708 PMC2945175

[B63] Nicholson G. Rantalainen M. Maher A. D. Li J. V. Malmodin D. Ahmadi K. R.. (2011). Human metabolic profiles are stably controlled by genetic and environmental variation. Mol. Syst. Biol. 7, 525. 10.1038/msb.2011.57 21878913 PMC3202796

[B64] Nicholson J. K. (2006). Global systems biology, personalized medicine and molecular epidemiology. Mol. Syst. Biol. 2, 52. 10.1038/msb4100095 17016518 PMC1682018

[B65] Nicholson J. K. Holmes E. Kinross J. Burcelin R. Gibson G. Jia W.. (2012). Host-gut microbiota metabolic interactions. Science 336, 1262–1267. 10.1126/science.1223813 22674330

[B66] Nicholson J. K. Lindon J. C. Holmes E. (1999). ‘Metabonomics’: understanding the metabolic responses of living systems to pathophysiological stimuli via multivariate statistical analysis of biological NMR spectroscopic data. Xenobiotica 29, 1181–1189. 10.1080/004982599238047 10598751

[B67] Palsson B. (2009). Metabolic systems biology. FEBS Lett. 583, 3900–3904. 10.1016/j.febslet.2009.09.031 19769971 PMC3119668

[B68] Palsson B. Zengler K. (2010). The challenges of integrating multi-omic data sets. Nat. Chem. Biol. 6, 787–789. 10.1038/nchembio.441 20976870

[B69] Patil K. R. Nielsen J. (2005). Uncovering transcriptional regulation of metabolism by using metabolic network topology. Proc. Natl. Acad. Sci. U.S.A. 102, 2685–2689. 10.1073/pnas.0406811102 15710883 PMC549453

[B70] Rezzi S. Collino S. Goulet L. Martin F. P. (2013). Metabonomic approaches to nutrient metabolism and future molecular nutrition. TrAC Trends Anal. Chem. 52, 112–119. 10.1016/j.trac.2013.09.00416537964

[B71] Richards S. E. Dumas M. E. Fonville J. M. Ebbels T. M. D. Holmes E. Nicholson J. K. (2010). Intra- and inter-omic fusion of metabolic profiling data in a systems biology framework. Chemometrics Intell. Lab. Syst. 104, 121–131. 10.1016/j.chemolab.2010.07.006

[B72] Romero P. Wagg J. Green M. L. Kaiser D. Krummenacker M. Karp P. D. (2005). Computational prediction of human metabolic pathways from the complete human genome. Genome Biol. 6:R2. 10.1186/gb-2004-6-1-r2 15642094 PMC549063

[B73] Rosenbloom A. L. Joe J. R. Young R. S. Winter W. E. (1999). Emerging epidemic of type 2 diabetes in youth. Diabetes Care 22, 345–354. 10.2337/diacare.22.2.345 10333956

[B74] Sandhu J. Ben-Shlomo Y. Cole T. J. Holly J. Davey Smith G. (2006). The impact of childhood body mass index on timing of puberty, adult stature and obesity: a follow-up study based on adolescent anthropometry recorded at Christ's Hospital (1936-1964). Int. J. Obes. (Lond.) 30, 14–22. 10.1038/sj.ijo.0803156 16344844

[B75] Scargle J. D. (1982). Studies in astronomical time series analysis. II-Statistical aspects of spectral analysis of unevenly spaced data. Astrophys. J. 263, 835–853. 10.1086/160554

[B76] Scargle J. D. (1989). Studies in astronomical time series analysis. III-Fourier transforms, autocorrelation functions, and cross-correlation functions of unevenly spaced data. Astrophys. J. 343, 874–887. 10.1086/167757

[B77] Scherer M. Montoliu I. Qanadli S. D. Collino S. Rezzi S. Kussmann M.. (2015). Blood plasma lipidomic signature of epicardial fat in healthy obese women. Obesity (Silver Spring). 23, 130–137. 10.1002/oby.20925 25400283

[B78] Schouteden M. Van Deun K. Pattyn S. Van Mechelen I. (2013). SCA with rotation to distinguish common and distinctive information in linked data. Behav. Res. Methods 45, 822–833. 10.3758/s13428-012-0295-9 23361416

[B79] Schusterova I. Leenen F. H. Jurko A. Sabol F. Takacova J. (2013). Epicardial adipose tissue and cardiometabolic risk factors in overweight and obese children and adolescents. Pediatr.Obes. 9, 63–70. 10.1111/j.2047-6310.2012.00134.x 23504985

[B80] Shen Y. Liu J. Estiu G. Isin B. Ahn Y. Y. Lee D. S.. (2010). Blueprint for antimicrobial hit discovery targeting metabolic networks. Proc. Natl. Acad. Sci. U.S.A. 107, 1082–1087. 10.1073/pnas.0909181107 20080587 PMC2824290

[B81] Shlomi T. Cabili M. N. Ruppin E. (2009). Predicting metabolic biomarkers of human inborn errors of metabolism. Mol. Syst. Biol. 5, 263. 10.1038/msb.2009.22 19401675 PMC2683725

[B82] Smith C. A. Want E. J. O'Maille G. Abagyan R. Siuzdak G. (2006). XCMS: Processing mass spectrometry data for metabolite profiling using nonlinear peak alignment, matching, and identification. Anal. Chem. 78, 779–787. 10.1021/ac051437y 16448051

[B83] Sommer F. Bäckhed F. (2013). The gut microbiota - masters of host development and physiology. Nat. Rev. Microbiol. 11, 227–238. 10.1038/nrmicro2974 23435359

[B84] Stanberry L. Mias G. I. Haynes W. Higdon R. Snyder M. Kolker E. (2013). Integrative analysis of longitudinal metabolomics data from a personal multi-omics profile. Metabolites 3, 741–760. 10.3390/metabo3030741 24958148 PMC3901289

[B85] Stanford N. J. Lubitz T. Smallbone K. Klipp E. Mendes P. Liebermeister W. (2013). Systematic construction of kinetic models from genome-scale metabolic networks. PLoS ONE 8:e79195. 10.1371/journal.pone.0079195 24324546 PMC3852239

[B86] Streeter A. J. Hosking J. Metcalf B. S. Jeffery A. N. Voss L. D. Wilkin T. J. (2013). Body fat in children does not adversely influence bone development: a 7-year longitudinal study (EarlyBird 18). Pediatr. Obes. 8, 418–427. 10.1111/j.2047-6310.2012.00126.x 23447431

[B87] Thakur A. (1991). Model: Mechanistic vs Empirical, in New Trends in Pharmacokinetics, eds Rescigno A. Thakur A. (New york, NY: Springer), 41–51. 10.1007/978-1-4684-8053-5_3

[B88] Thiele I. Price N. D. Vo T. D. Palsson B. O. (2005). Candidate metabolic network states in human mitochondria. Impact of diabetes, ischemia, and diet. J. Biol. Chem. 280, 11683–11695. 10.1074/jbc.M409072200 15572364

[B89] Tremaroli V. Bäckhed F. (2012). Functional interactions between the gut microbiota and host metabolism. Nature 489, 242–249. 10.1038/nature11552 22972297

[B90] Trygg J. Wold S. (2002). Orthogonal projections to latent structures (O-PLS). J. Chemom. 16, 119–128. 10.1002/cem.695

[B91] Trygg J. Wold S. (2003). O2-PLS, a two-block (X-Y) latent variable regression (LVR) method with an integral OSC filter. J. Chemom. 17, 53–64. 10.1002/cem.775

[B92] Wahl S. Yu Z. Kleber M. Singmann P. Holzapfel C. He Y.. (2012). Childhood obesity is associated with changes in the serum metabolite profile. Obes. Facts 5, 660–670. 10.1159/000343204 23108202

[B93] Walpole J. Papin J. A. Peirce S. M. (2013). Multiscale computational models of complex biological systems. Annu. Rev. Biomed. Eng. 15, 137–154. 10.1146/annurev-bioeng-071811-150104 23642247 PMC3970111

[B94] Westerhuis J. A. Hoefsloot H. C. J. Smit S. Vis D. J. Smilde A. K. Velzen E. J. J. (2008). Assessment of PLSDA cross validation. Metabolomics 4, 81–89. 10.1007/s11306-007-0099-6

[B95] Westerhuis J. A. Van Velzen E. J. J. Hoefsloot H. C. J. Smilde A. K. (2010). Multivariate paired data analysis: Multilevel PLSDA versus OPLSDA. Metabolomics 6, 119–128. 10.1007/s11306-009-0185-z 20339442 PMC2834771

[B96] Wikoff W. R. Anfora A. T. Liu J. Schultz P. G. Lesley S. A. Peters E. C.. (2009). Metabolomics analysis reveals large effects of gut microflora on mammalian blood metabolites. Proc. Natl. Acad. Sci. U.S.A. 106, 3698–3703. 10.1073/pnas.0812874106 19234110 PMC2656143

[B97] Wildman R. P. Muntner P. Reynolds K. McGinn A. P. Rajpathak S. Wylie-Rosett J.. (2008). The obese without cardiometabolic risk factor clustering and the normal weight with cardiometabolic risk factor clustering: prevalence and correlates of 2 phenotypes among the US population (NHANES 1999-2004). Arch. Intern. Med. 168, 1617–1624. 10.1001/archinte.168.15.1617 18695075

[B98] Wold S. Sjöström M. Eriksson L. (2001). PLS-regression: a basic tool of chemometrics. Chemometrics Intell. Lab. Syst. 58, 109–130. 10.1016/S0169-7439(01)00155-1

[B99] Yamakado M. Tanaka T. Nagao K. Ishizaka Y. Mitushima T. Tani M.. (2012). Plasma amino acid profile is associated with visceral fat accumulation in obese Japanese subjects. Clin. Obes. 2, 29–40. 10.1111/j.1758-8111.2012.00039.x 25586045

[B100] Yizhak K. Benyamini T. Liebermeister W. Ruppin E. Shlomi T. (2010). Integrating quantitative proteomics and metabolomics with a genome-scale metabolic network model. Bioinformatics 26, i255–i260. 10.1093/bioinformatics/btq183 20529914 PMC2881368

[B101] Zeger S. L. Liang K. Y. (1992). An overview of methods for the analysis of longitudinal data. Stat. Med. 11, 1825–1839. 10.1002/sim.4780111406 1480876

